# Health profile of captive African pygmy hedgehogs (*Atelerix albiventris*) in Paraná State, Southern Brazil

**DOI:** 10.1590/S1984-29612026030.

**Published:** 2026-07-20

**Authors:** Thiago Gallo Bizari, Flávia Carolina Meira Collere, André Saldanha Ferreira, Rogério Ribas Lange, Daiane Cristina Ribeiro Dambroski Nack, Maiara Paifer Martins Maia, Penélope Patricio Viviani de Moura, Rosangela Locatelli Dittrich, Fabiano Montiani Ferreira, Julia Galvão Arantes, Thállitha Samih Wischral Jayme Vieira, Rafael Felipe da Costa Vieira

**Affiliations:** 1 Universidade Federal do Paraná – UFPR, Hospital Veterinário, Centro de Ciências Agrárias, Departamento de Medicina Veterinária, Curitiba, PR, Brasil; 2 Universidade Federal do Paraná – UFPR, Departamento de Ciências Veterinárias, Palotina, PR, Brasil; 3 The University of North Carolina at Charlotte, Department of Chemistry, Charlotte, NC, USA; 4 The University of North Carolina at Charlotte, Center for Computational Intelligence to Predict Health and Environmental Risks – CIPHER, Charlotte, NC, USA; 5 The University of North Carolina at Charlotte, Department of Epidemiology and Community Health, Charlotte, NC, USA

**Keywords:** Hematological, biochemical, skin and fecal analyses, pathogens, polymerase chain reaction, Hematologia, bioquímica, análise fecal e cutânea, patógenos, reação em cadeia da polimerase

## Abstract

African pygmy hedgehogs (*Atelerix albiventris*) are omnivorous mammals native to sub-Saharan Africa and popular exotic pets worldwide. Although their trade is illegal, it remains common in Brazil. Therefore, the aims of this study were to investigate the health status of hedgehogs from illegal breeding sites, through hematological, biochemical and fecal analyses, skin pathogen screening and molecular assays for hemotropic *Mycoplasma* spp. and tick-borne pathogens. Real-time PCR assays for hemotropic *Mycoplasma* spp. (16S rRNA) and conventional PCR assays for *Theileria*/*Babesia* spp. (18S rRNA) and *Ehrlichia*/*Anaplasma* spp. (16S rRNA) were applied. No ectoparasites were found at the time of sampling, and hematological and biochemical results indicated a good health status. *Candida tropicalis* (15/25, 60%), *Mucor* spp. (12/25, 48%), and *Proteus vulgaris* (10/25, 40%) were identified in fecal analysis. Skin's microbiological examinations revealed *Staphylococcus* spp. (25/25 – 100%) and *Streptococcus* spp. (13/25 – 52%). All samples tested negative for hemotropic *Mycoplasma* spp., *Theileria*/*Babesia* spp., and *Ehrlichia*/*Anaplasma* spp. These findings suggest that the evaluated animals were not infected with harmful pathogens. Understanding their potential role in zoonotic diseases is necessary, particularly in the context of exotic species.

## Resumo

Os ouriços pigmeus africanos são mamíferos onívoros nativos da África subsaariana e populares como animais de estimação em todo o mundo. No Brasil, embora seu comércio seja ilegal, os confiscos continuam frequentes. Este estudo objetivou avaliar o estado de saúde de 25 ouriços de locais de criação ilegais, incluindo análises hematológicas e bioquímicas, exames microbiológicos fecais e cutâneos, e testes moleculares para *Mycoplasma* spp. hemotrópicos e patógenos transmitidos por carrapatos nesses animais. A PCR em tempo real foi usada para detectar o gene 16S rRNA de hemoplasmas, enquanto a PCR convencional visando os genes 18S rRNA e 16S rRNA foi usada para identificar *Theileria/Babesia* spp. e *Ehrlichia/Anaplasma* spp. Nenhum ectoparasito foi encontrado, e a avaliação hematológica e bioquímica indicou um bom estado de saúde. Nas fezes, o isolamento de *Candida tropicalis* (15/25, 60%), *Mucor* spp. (12/25, 48%) e *Proteus vulgaris* (10/25, 40%) foi proeminente, enquanto os exames microbiológicos cutâneos mostraram crescimento de *Staphylococcus* spp. (25/25, 100%) e *Streptococcus* spp. (13/25, 52%). Todas as amostras apresentaram resultados negativos para *Mycoplasma* spp., *Theileria*/*Babesia* spp. e *Ehrlichia*/*Anaplasma* spp. Os dados sugerem que os animais avaliados não estavam infectados por patógenos prejudiciais. Entender seu papel na transmissão zoonótica é necessário, principalmente se tratando de espécies exóticas.

African pygmy hedgehogs, *Atelerix albiventris* (Wagner, 1841) (Mammalia: Erinaceidae) are nocturnal, omnivorous mammals widely distributed across sub-Saharan Africa (Hutterer et al., 2005) but are considered an exotic species in Brazil. These animals are increasingly sought after as exotic pets (Ruszkowski et al., 2021), although they are illegal in several countries without special permits. Despite the prohibition of hedgehog trade and ownership in Brazil, confiscations remain frequent, underscoring the need for stronger policies to prevent uncontrolled breeding and spread.

Hedgehogs are commonly infested with various ectoparasites such as hard ticks (Acari: Ixodidae) and fleas (Sousa et al., 2006), which are recognized vectors of zoonotic pathogens including *Rickettsia* spp., *Borrelia* spp., *Ehrlichia* spp., *Anaplasma* spp., *Bartonella* spp., *Leishmania* spp., and tick-borne encephalitis viruses (Bezerra-Santos et al., 2021). The aims of this study were to investigate the presence of hemotropic *Mycoplasma*, selected tick-borne pathogens (TBP) and evaluate the hematological and biochemical profiles of African pygmy hedgehogs confiscated by the Água e Terra Institute (IAT) in Paraná State, southern Brazil.

The biological samples were obtained during a health assessment of the animals under the responsibility of the government agency (Setor de Fauna do Instituto Água e Terra - IAT), after being apprehended by the Environmental Military Force (Police Report No. 2021/58132). A total of 25 African pygmy hedgehogs were included in this study while under the custody of the State agency.

At the time of veterinary care, the animals were chemically restrained (ketamine 5 mg/kg and xylazine 1 mg/kg) for clinical examination and blood sampling. Blood samples (up to 3 mL) were collected by venipuncture using sterile EDTA-coated tubes (BD Vacutainer®, Franklin Lakes, NJ, EUA) and stored at –20 °C until molecular analysis. Additional samples (3 mL) were stored in tubes containing serum separator gel (BD Vacutainer®) and kept at room temperature (25 °C) until clot formation. The samples were then centrifuged at 1500 × g for 5 min, serum was separated, and aliquots were stored at − 20 °C for biochemical analysis.

The differential leukocyte count was performed by counting 100 leukocytes on a Giemsa-stained blood smear. Packed cell volume (PCV) was determined using the microhematocrit method with capillary tubes centrifuged at 11,000 rpm for 5 min (Farrand, 1976). Hemoglobin (Hgb) concentration was measured by the spectrophotometric cyanmethemoglobin method. Total red blood cell (RBC), white blood cell (WBC), platelet counts, and Hgb concentrations were measured with an automated hematology analyzer (BC-2800 Vet, Mindray®). Hematimetric indices, including mean corpuscular volume (MCV) and mean corpuscular hemoglobin concentration (MCHC), were calculated according to Wintrobe’s (1990) methodology.

Serum samples were thawed at room temperature and analyzed with an automated biochemical analyzer (BS-200, Myndray®), previously calibrated with a commercial control serum (Control Lab®). The following parameters were evaluated using the corresponding methods: total protein (biuret method, Katal®), albumin (bromocresol green method, Katal®), globulins (calculated as total protein minus albumin), urea (UV enzymatic urease-GLDH method, Kovalent®), creatinine (Jaffé method, Kovalent®), aspartate aminotransferase (AST; UV kinetic method, Katal®), alanine aminotransferase (ALT; UV kinetic method), and alkaline phosphatase (ALP, UV kinetic method).

Twenty-five fecal samples were collected from the coop, placed in sterile tubes and inoculated into tubes containing 2 mL of brain heart infusion (BHI) broth and incubated with shaking at 37 °C overnight. Aliquots were then plated on MacConkey agar plates supplemented with 2 μg/mL of ceftriaxone and colistin. Isolated colonies were subcultured on MacConkey agar plates without antibiotics and incubated at 37 °C for 18 h. Colonies were examined microscopically and identified morphologically (Winn et al., 2006).

Skin samples were individually collected from 25 animals by swabbing the interscapular region with sterile flexible rods. Swabs were plated on blood agar, and colonies were identified by light microscopy and biochemical tests following the methodology of Winn et al. (2006).

DNA from 200 μL whole blood was extracted using a commercial kit (ReliaPrepTM gDNA Blood Miniprep System, Promega, Madison, Wisconsin, USA), according to the manufacturer's instructions. Ultrapure water was used as a negative control to monitor cross-contamination. A conventional PCR (cPCR) assay targeting the mammalian endogenous gene glyceraldehyde-3-phosphate dehydrogenase (*gapdh*) was performed to ensure successful DNA extraction. Thereafter, hedgehog DNA samples were screened by a universal SYBR Green real-time PCR (qPCR) assay targeting the 16S rRNA gene of hemoplasmas (Willi et al., 2009). A standard curve was established using serial dilutions of gBlockTM (Integrated DNA Technologies, Coralville, IA, USA). Assays were conducted in accordance with the MIQE (Minimum Information for Publication of Quantitative Real-time PCR Experiments) guidelines (Bustin et al., 2009). Moreover, DNA samples from hedgehogs were also screened by conventional PCR assays targeting a fragment (551 bp) of the 18S rRNA gene of *Theileria*/*Babesia* spp. (Almeida et al., 2012) and a fragment (349 bp) of the 16S rRNA gene of *Ehrlichia*/*Anaplasma* spp. (Parola et al., 2000). DNA from *Babesia vogeli* and *Ehrlichia canis* obtained from naturally infected dogs and nuclease-free water were used as positive and negative controls, respectively.

The chi-square test was used to evaluate deviations from reference hematological values. A 95% confidence interval and p-values were calculated (Tables 1 and 2). Most data follow descriptive statistical behavior and is presented directly. Data were compiled and analyzed using Microsoft ExcelTM and the MedCalc® software.

**Table 1:** Summary statistics analyses of hematological profile of Captive *Atelerix albiventris* (Mammalia: Erinaceidae) in Paraná State, southern Brazil.

**Analyte**	**UNIT**	**N**	**Mean**	**SD**	**Median**	**Min**	**Max**	**p-value** *
**RBC**	10^12^/L	25	5.9	0.99	5.68	4.3	7.43	0.2
**PCV**	%	24	40.70	4.64	40.5	33	53	0.1
**Hemoglobin**	g/L	24	120.17	18.68	118.5	92	161	0.02
**WBC**	10^6^/L	17	12.3	5.1	12	6	21.8	0.01
**Neutrophils**	%	17	52.29	12.5	53	32	72	-
**Bands**	%	17	0.29	0.58	0	0	2	-
**Lymphocytes**	%	17	28.9	9.91	30	16	47	-
**Monocyte**	%	17	6.35	4.13	5	1	18	-
**Eosinophils**	%	17	9.94	5.91	9	0	19	-
**Basophils**	%	17	1.70	1.72	1	0	6	-
**Neutrophils**	10^6^/L	17	6.58	3.37	5.73	1.92	13.46	0.7
**Bands**	10^6^/L	17	0.043	0.1	0	0	0.38	0.02
**Lymphocytes**	10^6^/L	17	3.47	1.8	3.36	1.08	8.93	0.6
**Monocyte**	10^6^/L	17	0.78	0.6	0.67	0.12	2.25	0.4
**Eosinophils**	10^6^/L	17	1.16	0.84	0.93	0	3.4	0.3
**Basophils**	10^6^/L	17	0.21	0.22	0.18	0	0.88	0.1

Note: UNIT = unit of measurement; N = sample size; SD = standard deviation.

* p-value calculated by chi-square test.

**Table 2:** Summary statistics analyses of serum biochemistry profile of captive *Atelerix albiventris* (Mammalia: Erinaceidae) in Paraná State, southern Brazil.

**ANALYTE**	**UNIT**	**N**	**MEAN**	**SD**	**MEDIAN**	**MIN**	**MAX**	**P-VALUE** *
**UREA**	mmol/L	22	4.20	0.95	4.04	2.44	6.79	0.08
**CREATININE**	mmol/L	22	40.99	8.43	44.21	26.52	53.05	0.6
**ALP**	U/L	22	64.97	27.41	57.9	10.1	133.9	0.05
**ALT**	U/L	21	67.22	23.89	60.6	32.9	120.7	<0.01
**AST**	U/L	21	29.76	14.22	28.6	14.3	80.4	<0.01
**TOTAL PROTEIN**	g/L	23	69	13.54	69	47	113	0.9
**ALBUMIN**	g/L	23	26.34	5.17	27	10	25	<0.01
**GLOBULIN**	g/L	23	43.04	17.46	41	20	112	0.9
**A:G RATIO**		23	0.69	0.25	0.64	0.08	1.35	

Note: UNIT = unit of measurement; N = sample size; SD = standard deviation.

* p-value calculated by chi-square test.

The mean (x̅) and standard deviation (s) of the RBC count and hematocrit were 5.9± 0.99x10^12^/L and 40.7:4.64%, respectively. Summary statistics analyses of hematological and biochemical results are presented in Tables 1 and 2. Some samples were excluded from WBC counts and differential analyses due to clot formation. Additionally, samples with insufficient volume to repeat or confirm biochemical tests were excluded.

The hematological values of African pygmy hedgehogs in this study showed greater variation than those reported in previous studies (Okorie-Kanu et al., 2015). Although all animals were clinically healthy, some cases of leukocytosis were observed, suggesting possible inflammatory activity. Interpretation of data was limited by the difficulty of determining the animals’ ages and the scarcity of reference values for this species. However, the red blood cell count (5.9 ± 0.99 × 10^12^/L) was slightly lower than the values reported in the literature for healthy captive animals (7.5 to 9.5 × 10^12^/L) (Okorie-Kanu et al., 2015). This discrepancy may be due to physiological adaptations resulting from poor nutritional management in illegal breeding operations or to individual variations within the Brazilian hedgehog lineage.

Serum biochemical analyses also differed from earlier reports on this species or similar ones (Rossi et al., 2014, Okorie-Kanu et al., 2015). Compared with domestic animals, elevated AST values in one hedgehog may indicate liver or muscle injury, especially given the concurrent increase in CK activity. Similarly, the mild hypoalbuminemia observed suggests an inflammatory process, since albumin is a negative acute-phase protein. During inflammation, hepatocytes increase the synthesis of specific acute-phase globulins, while B lymphocytes produce immunoglobulins, leading to a relative decrease in albumin levels.

From the 25 fecal samples, the isolates (Table 3) included *Candida tropicalis* (15/25, 60%, 95% CI: 0,40-0,76), *Mucor* spp. (12/25, 48%, 95%, CI: 0,30-0,66), *Proteus vulgaris* (10/25; 40%, 95% CI: 0,23-0,59), and *Enterobacter sakazakii* (seven/25; 28%, 95% CI: 0,14-0,47) were most frequent, followed by *Proteus mirabilis* (four/25; 16%, 95% CI: 0,06-0,34), *Citrobacter amalonaticus* (two/25; 8%, 95% CI: 0,02-0,24), *Enterobacter* spp. (two/25; 8%, 95% CI: 0,02-0,24), *Geotrichum* spp. (one/25, 4%, 95% CI: 0,01-0,19), and *Aspergillus* spp. (one/25, 4%, 95% 0,01-0,19). Fecal analysis differed from previous studies (Amanbayeva et al., 2021), likely due to geographic variation and its effect on the microbiota.

**Table 3:** Absolute frequency, prevalence, and confidence interval (95% CI) of pathogens isolated from the feces and skin of the *Atelerix albiventris* (Mammalia: Erinaceidae) in Paraná State, southern Brazil.

**SAMPLE**	**PATHOGEN**	**FREQUENCY (N)**	**PREVALENCE (%)**	**95% CI**
**FECES**	*Candida tropicalis*	15	60%	0.40 – 0.76
	*Mucor* spp.	12	48%	0.30 – 0.66
	*Proteus vulgaris*	10	40%	0.23 – 0.59
	*Enterobacter sakazakii*	7	28%	0.14 – 0.47
	*Proteus mirabilis*	4	16%	0.06 – 0.34
	*Citrobacter amalonaticus*	2	8%	0.02 – 0.24
	*Enterobacter* spp.	2	8%	0.02 – 0.24
	*Geotrichum* spp.	1	4%	0.01 – 0.19
**SKIN SWAB**	*Staphylococcus* spp.	25	100%	0.86 – 1.00
	*Streptococcus* spp.	13	52%	0.33 – 0.69
	*Bacillus* spp.	4	16%	0.06 – 0.34
	*Micrococcus* spp.	2	8%	0.02 – 0.24

*Salmonella* spp. has been reported in *A. albiventris* (Perez et al., 2021), raising concern about its role in transmission. However, this bacterium was not detected in the present study, which may again reflect the environment in which these animals were housed. Although not performed, fecal parasitological examinations could provide information regarding the intestinal parasitic microbiota of these animals and reveal further epidemiological data.

In the microbiological examinations of fecal samples, the detection of *Candida* spp. in 60% of animals is noteworthy, given the zoonotic potential of this pathogen. This finding is particularly relevant for direct handlers, such as zookeepers and veterinarians, and for individuals with compromised immune systems. It is important to note that these microbial isolates were identified using conventional phenotypic and morphological methods. While these techniques have limitations in terms of taxonomic resolution at the species level compared to molecular biology or proteomics methods, they are essential for initial microbiological diagnosis. Subsequent studies using genetic sequencing techniques are recommended to refine the taxonomic classification and investigate the phylogenetic diversity of microorganisms associated with this exotic species. Furthermore, specific species may be parasitizing these animals, and there may be a variety of agents in common with other potential contacts.

Four pathogens were detected parasitizing the skin (Table 3), with some animals exhibiting co-infections. *Bacillus* spp. (four/25; 16%, 95% CI: 0,06-0,34), *Micrococcus* spp. (two/25; 8%, 95% CI: 0,02-0,24); *Staphylococcus* spp. (25/25; 100%, 95% CI: 0,86-1,0), and *Streptococcus* spp. (13/25; 52%, 95% CI: 0,33-0,69).

On the other hand, the detection of *Staphylococcus* spp. in all skin samples and *Streptococcus* spp. in 52% of samples indicates their consistent presence in the skin. These organisms may act as opportunistic pathogens under dysbiosis conditions and may also pose a risk to conspecifics and other immunocompromised animals.

Other identified pathogens may act as secondary opportunistic agents in skin or systemic diseases, since they appear to be part of the microbiota. In Indonesia, vaginal swabs from some hedgehogs revealed *Proteus* spp. and *E. coli* (Ash-Santri et al., 2021). Compared with this study, a greater variety of agents were detected in the skin than in the genital tract.

The *gapdh* gene was successfully amplified in all hedgehog samples. All samples tested negative for hemoplasmas, *Ehrlichia*/*Anaplasma* spp., and *Theileria*/*Babesia* spp. Hedgehogs are considered potential hosts or reservoirs of zoonotic vector-borne pathogens (Krawczyk et al., 2015) and are frequently parasitized by ticks and mites (Iacob & Iftinca, 2018). Ticks commonly associated with hedgehogs including species of the genera *Amblyomma*, *Rhipicephalus*, *Dermacentor*, *Hyalomma* (Ixodidae), and *Ornithodoros* (Argasidae) (Estrada-Peña et al., 2018).

Although most animals appeared clinically healthy, the leukocytosis observed in specific individuals suggests an active inflammatory response. Given the negative results of molecular tests for blood parasites, this finding may be related to secondary opportunistic infections caused by the identified *Staphylococcus* and *Streptococcus* isolates (Girling & Heatley, 2020) or chronic stress resulting from post-capture husbandry conditions. Chronic stress is known to modulate the leukocyte profile in small wild mammals (Okorie-Kanu et al., 2015).

Wild animals, mainly those with synanthropic behavior, are important reservoirs of zoonotic pathogens (Hassell et al., 2017). In Brazil, studies have identified tick-borne pathogens in synanthropic species, some of considerable zoonotic relevance (Orozco et al., 2022). In a previous study, *A*. *phagocytophilum* and *Borrelia* spp. were detected in ticks collected from European hedgehogs (*Erinaceus europaeus*) (Krawczyk et al., 2015).

Although various zoonotic pathogens, including bacteria, viruses, protozoa, and fungi, have been reported in hedgehogs (Krawczyk et al., 2015), all animals in the present study tested negative for hemoplasmas and tick-borne pathogens. Although the animals' good health, as evidenced by their hematological and biochemical parameters, suggests a low parasite load, the absence of specific coproparasitological analyses limits the detection of gastrointestinal endoparasites. Therefore, a direct diagnosis is necessary to exclude helminths or protozoa with zoonotic potential or low virulence. This reinforces the need for more comprehensive diagnostic protocols in subsequent studies involving seized animals.

Due to their exotic nature in Brazil, it is not possible to accurately determine the behavior of these animals when epidemiologically challenged by pathogens found within our territory. Furthermore, the scarcity of studies on this species in our country raises greater concern regarding potential risks.

Hedgehogs thrive in urban, rural, and natural environments in close contact with domestic animals and humans (Skuballa et al., 2010), and their introduction into new ecosystems can cause ecological disturbance. Because the animals in this study were seized from captive breeding facilities, they may not have been exposed to arthropods capable of transmitting pathogens. In general, animals bred for trade are more likely to have a controlled health status. This could explain the absence of pathogens and ectoparasites in the studied animals. It should be noted that the sampling in the present study is limited, as the animals originated from police seizures, which makes it difficult to infer these results to other populations under different environmental conditions.

Although the hematological and biochemical data indicate good health, mild leukocytosis in some individuals suggests a subclinical inflammatory response possibly caused by handling and the stress of illegal captivity. The absence of blood parasites suggests relative isolation from wild vectors, however, the detection of agents with pathogenic potential in asymptomatic animals reinforces the need for rigorous health protocols in cases of seizure.

Furthermore, the diversity of the identified cutaneous and fecal microbiota, including the presence of opportunistic pathogens, underscores *A. albiventris*'s potential as a reservoir for microorganisms with zoonotic relevance. Therefore, expanding scientific knowledge of the hematological and biochemical profiles of *A*. *albiventris*, as well as the diseases affecting this species, is essential to improve understanding of its health status and their role in zoonotic transmission.

The findings of this study reiterate that apparent health does not negate the potential spread of microorganisms between exotic animals and humans. From a One Health perspective, epidemiological surveillance of these hosts is essential for wildlife conservation and as a strategic public health measure to mitigate risks arising from contact with pathogenic microbiota in captive and seizure settings.

## Data Availability

The data generated during the study are included in this article.
